# Addressing inter-device variations in optical coherence tomography angiography: will image-to-image translation systems help?

**DOI:** 10.1186/s40942-023-00491-8

**Published:** 2023-08-29

**Authors:** Hosein Nouri, Reza Nasri, Seyed-Hossein Abtahi

**Affiliations:** 1 Ophthalmic Research Center, Research Institute for Ophthalmology and Vision Science, Shahid Beheshti University of Medical Sciences, Tehran, Iran; 2 School of Medicine, Isfahan University of Medical Sciences, Isfahan, Iran; 3 School of Engineering, University of Isfahan, Isfahan, Iran; 4 Department of Ophthalmology, Torfe Medical Center, Shahid Beheshti University of Medical Sciences, Tehran, Iran

**Keywords:** Optical coherence tomography angiography, Artificial Intelligence, Generative Adversarial Network, Denoising Diffusion Probabilistic Model, Unsupervised machine learning, Deep learning

## Abstract

**Background:**

Optical coherence tomography angiography (OCTA) is an innovative technology providing visual and quantitative data on retinal microvasculature in a non-invasive manner.

**Main body:**

Due to variations in the technical specifications of different OCTA devices, there are significant inter-device differences in OCTA data, which can limit their comparability and generalizability. These variations can also result in a domain shift problem that may interfere with applicability of machine learning models on data obtained from different OCTA machines. One possible approach to address this issue may be unsupervised deep image-to-image translation leveraging systems such as Cycle-Consistent Generative Adversarial Networks (Cycle-GANs) and Denoising Diffusion Probabilistic Models (DDPMs). Through training on unpaired images from different device domains, Cycle-GANs and DDPMs may enable cross-domain translation of images. They have been successfully applied in various medical imaging tasks, including segmentation, denoising, and cross-modality image-to-image translation. In this commentary, we briefly describe how Cycle-GANs and DDPMs operate, and review the recent experiments with these models on medical and ocular imaging data. We then discuss the benefits of applying such techniques for inter-device translation of OCTA data and the potential challenges ahead.

**Conclusion:**

Retinal imaging technologies and deep learning-based domain adaptation techniques are rapidly evolving. We suggest exploring the potential of image-to-image translation methods in improving the comparability of OCTA data from different centers or devices. This may facilitate more efficient analysis of heterogeneous data and broader applicability of machine learning models trained on limited datasets in this field.

## Background

Optical coherence tomography angiography (OCTA) is an innovative, non-invasive technology capable of visualizing the flow within the retina. With its ability to provide quantitative data on retinal microvasculature at different levels, OCTA holds great promise for facilitating the diagnosis and management of various retinal disorders [1]. The rapid, continuous advancements in artificial intelligence (AI) and deep learning (DL) have potentiated several applications of computer vision in medical image analysis, including image classification, object detection, and segmentation [2]. However, inter-device variations in quantitative OCTA data [3, 4] may limit the applicability of DL models across different OCTA devices – not to mention its restricting effect on the comparability of data from various studies and establishment of normative/disease-specific value ranges.

## Main text

### Inter-device variations in technical details and outputs

**Fig. 1:**
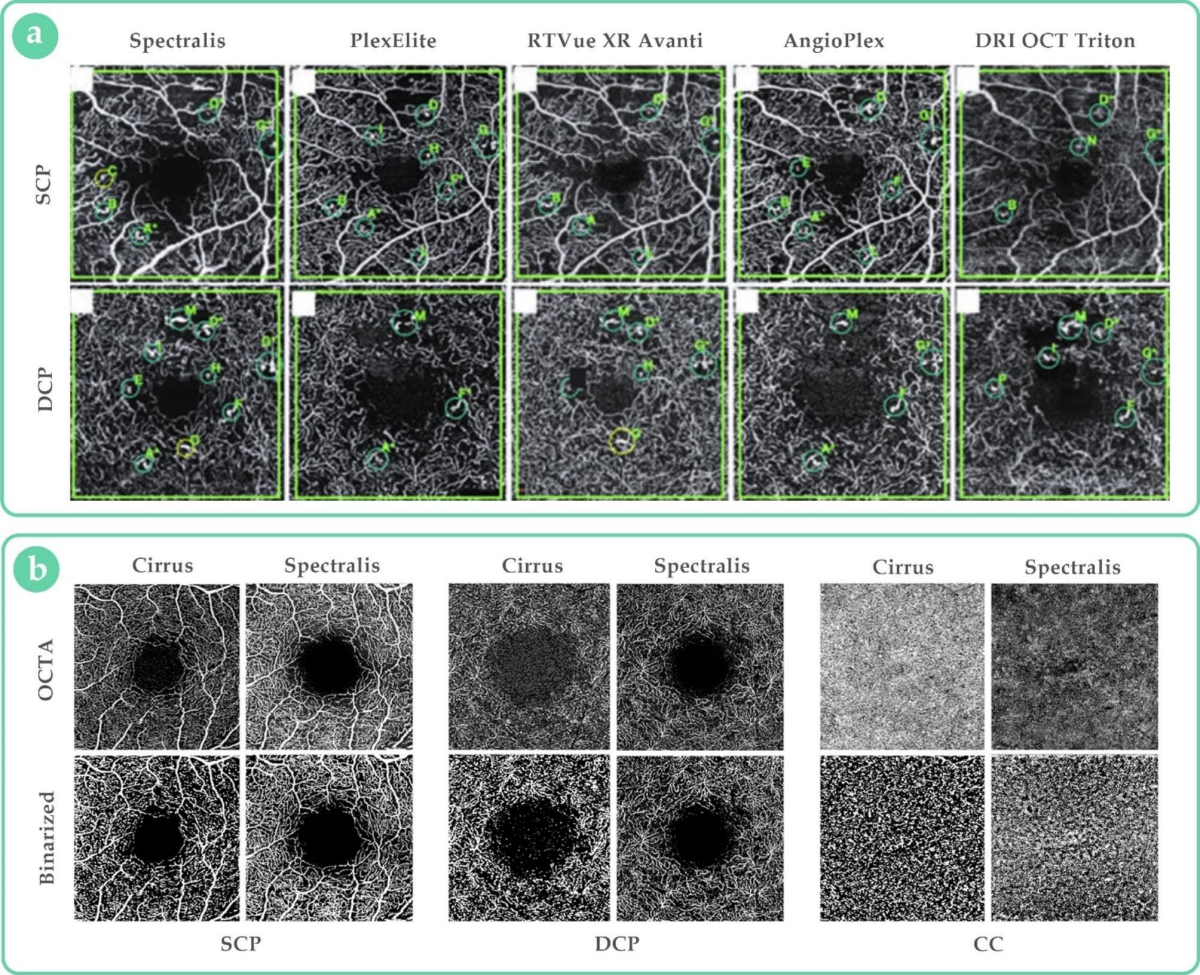
Comparison of optical coherence tomography angiography (OCTA) images from different devices. **(a)**: en face scans by five commercial devices at the level of superficial and deep capillary plexuses (SCP and DCP) – small circles represent the detectable microaneurysms, denoted by alphabetical letters [4]; **(b)**: the upper row shows en face scans at the level of SCP, DCP, and choriocapillaris (CC) obtained by two machines; the lower row shows corresponding binarized images [6]

Several comparative studies have highlighted inconsistencies in the information obtained from different OCTA machines. Parrulli and colleagues found significant inter-device variability in the visualization of microaneurysms secondary to diabetic retinopathy (DR) using five OCTA machines. The mean numbers of detectable microaneurysms per eye, in both superficial and deep plexuses, were 16.1 ± 6.4 (Spectralis), 10.2 ± 4.5 (PlexElite), 9.3 ± 3.4 (RTVue XR), 8.1 ± 3.8 (AngioPlex), and 8.9 ± 3.5 (DRI OCT Triton) (Fig. 1.a) [4].

In addition to such visual variations, inter-device differences in quantitative measurements have also been reported. Corvi et al. found significant differences in the area, vessel density, and fractal dimension of choroidal neovascularizations when using four different devices (PlexElite, Spectralis, RTVue XR, and AngioPlex). The Bland-Altman analysis indicated that differences between devices were too large to regard their measurements as interchangeable in routine practice [5]. Moreover, results from a study on 80 eyes with different DR severities showed poor agreement between Zeiss and Heidelberg OCTA devices in nearly all quantitative parameters except superficial vessel density – i.e., superficial vessel length density, deep vessel density, deep vessel length density, and choriocapillaris flow voids (Fig. 1.b) [6]. The presence or severity of retinopathy did not affect the magnitude of discrepancy [6]. The measurement of foveal avascular zone (FAZ) may also be subject to such inter-device inconsistencies, as reported by Anvari and coworkers; the mean FAZ area (mm^2^) was 0.31 ± 0.08 (AngioVue) and 0.55 ± 0.16 (Spectralis) (p < 0.001) at the superficial layer and 0.26 ± 0.08 (Optovue) and 0.36 ± 0.13 (Spectralis) (p < 0.001) at the deep layer [7].

These variations have been mostly attributed to differences in technical specifications of commercial devices and their decorrelation and segmentation algorithms [4–7]. In brief, the operating principle of OCTA involves comparing repeated B-scans of the same retinal section pixel-by-pixel to spot motion contrast corresponding to detectable vascular flow [8]. The sensitivity of an OCTA device is primarily determined by the inter-scan time – the shorter the inter-scan time, the more likely it is to miss areas with slower flow, and vice versa. The inter-scan time consists of the following elements, which may differ from one device to another:

i) the B-scan acquisition time: depends on the A-scan rate (e.g., 70,000/s in AngioVue, 85,000/s in Spectralis, and 100,000/s in PlexElite) and sampling density (e.g., 304 × 304 in AngioVue, 512 × 512 in Spectralis, and 300 × 300 in PlexElite – for a 3 × 3 scan).
ii) the fly-back time: refers to the time it takes for the OCT beam to return from one end of the B-scan to the other to repeat the same B-scan [8].

Even after receiving decorrelation signals, different signal processing methods can further contribute to inter-device variability of the output data. For instance, a higher level of threshold masking in one device – aimed at reducing noise – may inadvertently conceal some weak yet present signals in the output, resulting in an attenuation artifact [8]. Further heterogeneities may also result from each device’s built-in segmentation algorithm and boundaries [4].

### Domain shift and domain adaptation

These inter-device output discrepancies limit the comparability and generalizability of OCTA data and can also be considered a form of ‘domain shift’ problem. In the context of machine learning (ML), domain shift refers to changes in data distribution between the training dataset (source domain) and the test dataset (target domain). Here, domain refers to the specific device type and acquisition protocol, and domain shift reflects the differences in imaging devices and their parameterization [9].

Domain shift can significantly impair the performance of ML algorithms or DL models when applied to new, unseen data with different distributions from their training dataset. For example, a DL model that performs well on magnetic resonance imaging (MRI) data from one center may exhibit a weaker performance on MRI scans from another center [10].

Numerous domain adaptation methods have been developed and applied to address the challenge of domain shift when using ML/DL models with real-world data. In the context of medical image analysis, domain adaptation (DA) methods can be classified based on the model type (shallow/deep), label availability (supervised/semi-supervised/unsupervised), and modality difference (single-source/multi-source), among other factors. A comprehensive discussion on various DA techniques is available elsewhere for the interested readership [10].

### Image-to-image translation methods

Unsupervised deep image-to-image translation is a suitable domain adaptation method for our subject problem. An advantage of unsupervised training is not requiring paired image samples; obtaining exactly paired OCTA images using different devices can be technically difficult, expensive, and time-conuming. The main concept of image-to-image translation involves mapping the features of an image from a source domain to the style of a target domain in a content-preserving manner [11]. Following is a brief overview of two systems we find helpful for this purpose – i.e., Cycle-consistent Generative Adversarial Network (Cycle-GAN) [12] and Denoising Diffusion Probabilistic Model (DDPM; also called the “diffusion model” for brevity) [13].

#### Cycle-consistent generative adversarial network

Cycle-GAN was first introduced by Zhu and colleagues [12] as a variant of the original GAN architecture [14]. Cycle-GANs perform unpaired image-to-image translation via two types of systems working together in an adversarial manner:

i) ‘generator’ for synthesizing images.
ii) ‘discriminator’ for discerning actual images from synthetic ones.

For example, let there be two OCTA devices, ‘source’ and ‘target’, and let us call images from (or in style of) the source and target devices as A and B, respectively. The original Cycle-GAN incorporates two of each type of system [12]:

- Generator 1 [G]: given A, returns synthetic B.
- Discriminator 1: discerns real B from synthetic B.
- Generator 2 [F]: given B, returns A.
- Discriminator 2: discerns real A from synthetic A.

**Fig. 2:**
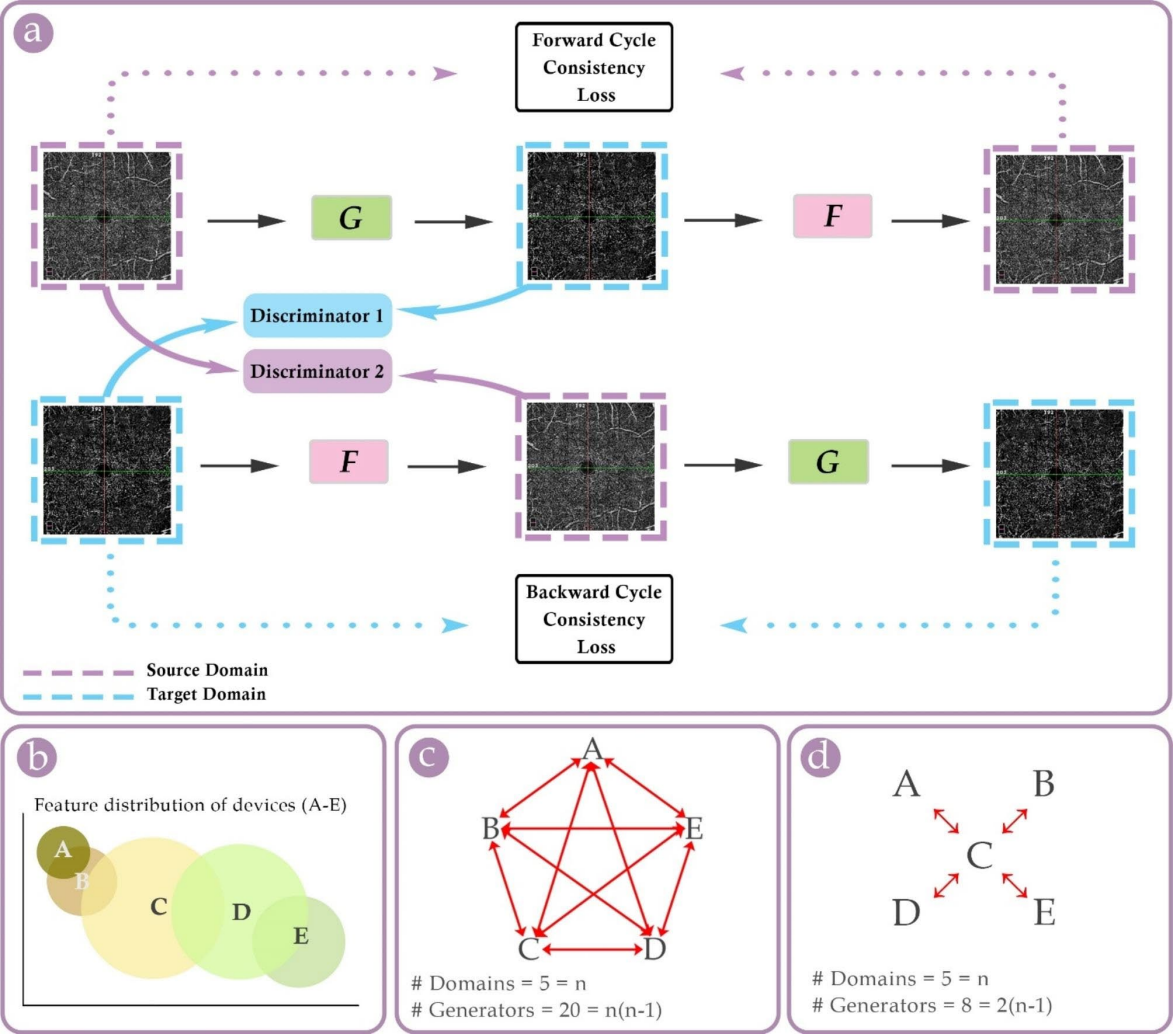
**(a)**: Illustration of a cycle-consistent generative adversarial network (Cycle-GAN) – dashed squares in purple and cyan denote images from (or in the style of) device A (source domain) and device B (target domain), respectively; ***G*** represents Generator 1 [source image → target image], and ***F*** represents Generator 2 [target image → source image]; **(b)**: schematic map of hypothetical feature distributions of five devices; the actual feature maps are presumably more overlying, but for illustrative purposes, they are located more distantly in this illustration; **(c)**: conventional direct cross-device translations diagram; **(d)**: indirect cross-device translations using an intermediate domain as the bridge; **Note**: every two generators in one Cycle-GAN (i.e., forward and backward) are denoted by a red bidirectional arrow

For translation consistency, each synthetic B produced by Generator 1 is fed to Generator 2 to produce a synthetic A – forming a ‘cycle’. This synthetic A is compared pixel-by-pixel to the actual A, and the error (cycle-consistency loss) is calculated and minimized over each epoch. A similar process is also done for the other generator system (see Fig. 2.a). In the meantime, the error of each discriminator in differentiating actual images from fake ones is gradually minimized by training.

Thus, both generators are continuously trained to synthesize such realistic images that can fool the discriminators, whose accuracy is also improving simultaneously. We depicted a schematic visualization of the Cycle-GAN framework (see Fig. 2.a). To clarify, we used the word ‘image’ and inserted a 2D OCTA scan in Fig. 2, but it should not mislead to the assumption that the input/output of Cycle-GANs are limited to 2D pictures – a tensor object like an OCTA-scanned cube would be suitable as well.

#### Denoising diffusion probabilistic model

**Fig. 3:**
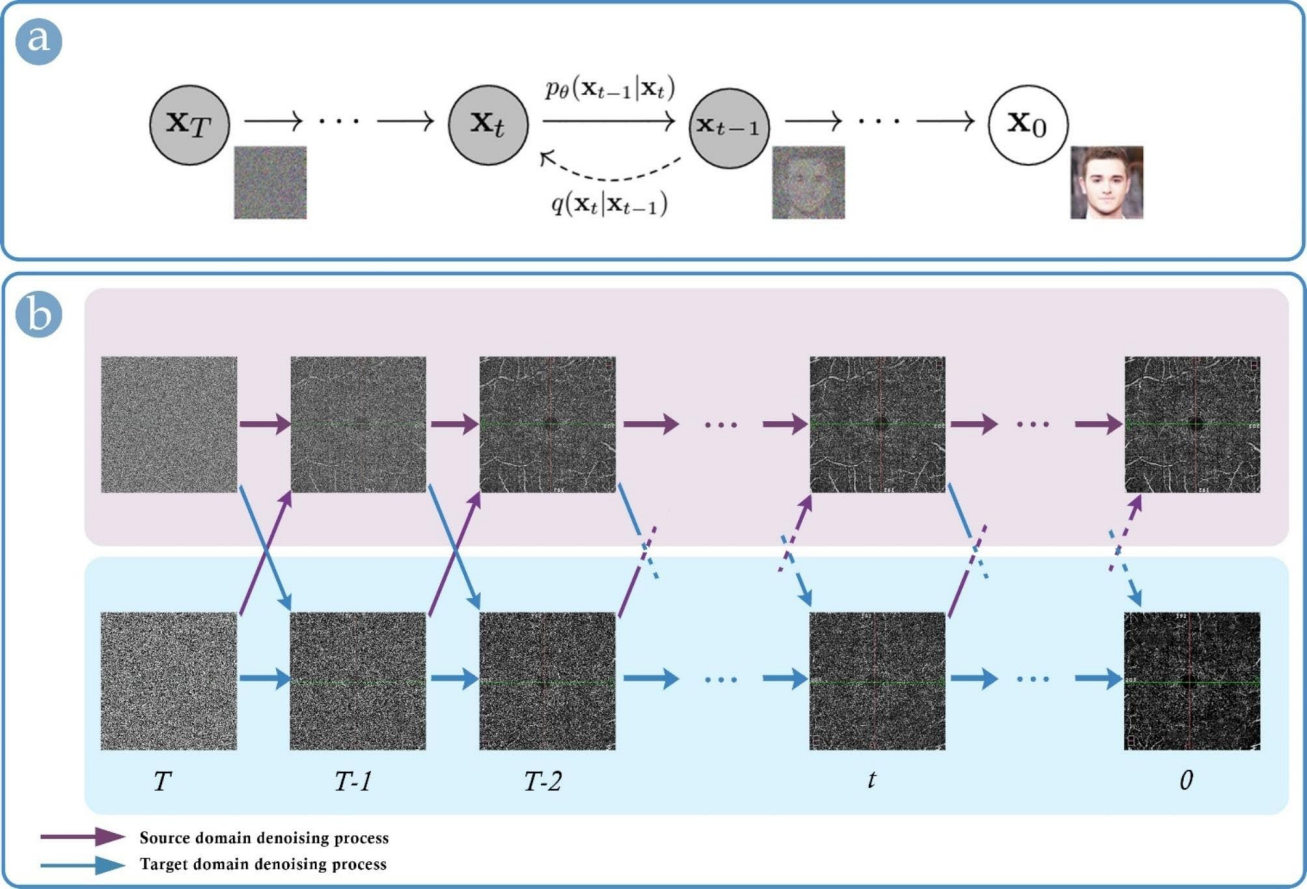
**(a)**: the original graphical depiction of the Denoising Diffusion Probabilistic Model by Ho et al., representing a denoising Markov Chain with ***T*** steps – for a given noisy sample ***t***, a neural network is trained to approximate the next denoised image ***t-1*** through calculating a probabilistic distribution of denoised data [13]; **(b)**: a customized illustration of the conceptual framework of UNIT-DDPM, proposed by Sasaki et al. [15]

Recent years have also witnessed parallel lines of research on non-adversarial generative networks that could enhance the quality of image-to-image translation. One such system with higher similarity scores than GAN is DDPM [13]. Throughout thousands of consecutive steps (i.e., Markov Chain), a diffusion model progressively adds Gaussian noise to an actual image and, then, as a generative model, reverses the corruption and tries to learn thousands of small steps to reconstruct high-quality, noise-free images from nearly pure noise (Fig. 3.a) [13]. Despite their time-consuming training process, diffusion models are becoming increasingly popular in light of their high-quality outputs.

While diffusion models are most frequently utilized for *de novo* image synthesis, in 2021, Sasaki et al. proposed a novel approach based on DDPM, i.e., UNpaired Image Translation with DDPM (UNIT-DDPM) [15]. The conceptual framework of UNIT-DDPM is illustrated (Fig. 3.b). Their approach connects the latent information learned by diffusion models across different domains, allowing the whole system to learn to gradually transform a pure noise sample to an image in the style of the target domain in a way that is related to the input image from the source domain. Their method brought about ~ 20% improvement in the accuracy of several state-of-the-art image-to-image translation models, including Cycle-GAN [15].

### Applications of image-to-image translation in medical and ocular imaging

#### Cycle-GANs

Many teams have investigated the utilization of Cycle-GANs for unpaired medical image-to-image translation, serving a variety of purposes, such as segmentation, contouring, denoising, and cross-modality translation – reviewed in detail by Chen et al. [2]. To name a few, some studies have utilized or modified the Cycle-GAN structure to:

i) transform computed tomography (CT) scans to MR images and vice versa [16],
ii) translate one MRI modality to another (e.g., proton density-weighted to T2-weighted) [17],
iii) correct CT scans affected by metal artifacts [18], or.
iv) reconstruct full-dose CT images from low-dose counterparts [19].

Moreover, applications of Cycle-GANs in transforming ophthalmic imaging data for the following purposes have also garnered attention [20]:

i) Data augmentation – e.g., synthesizing realistic images of rare diseases based on non-diseased counterparts to provide additional training data for diagnostic DL models’ training:
  - normal OCT images → OCT images of rare retinal diseases, like macular telangiectasia, retinitis pigmentosa, macular hole, etc. [21].
  - normal ocular surface images → ocular surface images of conjunctival melanoma [22].
ii) Image enhancement – e.g., denoising, super-resolution, and artifact reduction:
  - noisy/artifactual [23] or hazy cataractous [24] color fundus photographs (CFP) → denoised [23] or dehazed [24] CFPs.
  - noisy/low-resolution retinal OCT images → denoised high-resolution retinal OCT images – outperforming several state-of-the-art denoising algorithms [25, 26].
iii) Domain transfer – e.g., cross-modality translation of images:
  - Ultra-widefield fundus photographs → traditional CFPs [27] – and vice versa [28].

Among works involving OCT data is the study by Manakov and colleagues, who modified the Cycle-GAN to reconstruct low-noise structural OCT scans (60 frames) from high-noise inputs (12 frames) (task ii.b). Their model outperformed some state-of-the-art denoising algorithms quantitatively and qualitatively (per masked ophthalmologists’ evaluation) and required 30% less computational power. A novelty of their work was using a shared discriminator instead of two, which allowed their model to focus on learning subtle inter-domain differences instead of learning redundant domain features that were identical in both domains. This technique also significantly enhanced the quality of the generated images [25]. Such innovation may be relevant to our problem, as discussed later.

Differences in structural OCT images from different machines can degrade the performance of DL models for retinal layer segmentation [29, 30]. To overcome this obstacle, Romo-Bucheli et al. utilized Cycle-GAN to translate OCT images from Cirrus to those from Spectralis [30]. This maneuver allowed their segmentation models, trained on images from each device, to maintain accuracy when applied to images from the other device [30].

#### Diffusion models

The performance of diffusion models in several medical imaging tasks involving image generation, denoising, reconstruction, segmentation, and translation is being actively explored. However, their applications in this field are in the early stages, and there is limited available experience, compared to GANs [31]. Promising results have been reported by the few studies that employed diffusion models for cross-modality medical image translation (e.g., CT-to-MR images), outperforming GANs [31].

In the field of ophthalmology, most of the existing experience involves *de novo* synthesis of images, like CFPs (i.e., data augmentation for more efficient training of DL models), in which the DDPM approach appears to do better than the original GAN method [32]. More recently, Hu and associates experimented with DDPM to denoise speckled OCT images so that the model would only need to learn the speckle pattern rather than the entire retinal structural details [33]. Their proposed method resulted in clearer visualization of subtle elements, like external limiting membrane; it also improved the signal-to-noise ratio more than the conventional method of averaging multiple b-scans at the same position [33].

### Cross-device OCTA image translation

Efficient integration of detailed OCTA data in clinical practice requires addressing several issues, among which are the described variations in visual and quantitative outputs of different devices (see section “Inter-device variations in technical details and outputs”). These variations can make it difficult to standardize value ranges – normal or disease-specific. Furthermore, manual processing and analysis of complex 3D OCTA data may be labor-intensive, justifying the development of DL-based models for such tasks [34]. Yet, the domain shift problem can seriously affect the robustness of such DL models trained on data from a limited number of machines – as described earlier.

We assume that inter-device differences in OCTA data may be more concerning than in structural OCT data. Different OCT domains may show variations in the measured thickness of retinal layers, which may be less confusing to the interpreting ophthalmologist. Nevertheless, such differences are significant [35] and, as mentioned earlier, can degrade the performance of DL models on unseen data [29, 30]. With OCTA data, not only do inter-device variations limit the generalizability of DL models, but they are also more puzzling to interpret at the clinical level. That may be because:

i) Quantitative OCTA metrics are more diverse than quantitative structural OCT measures.
ii) OCTA data is a derivative of OCT data, meaning there are two levels of inter-device variations; one is related to the acquisition and processing of structural data, and the other involves the technical aspects of flow signal mapping (as described in section “Inter-device variations in technical details and outputs”).

To that end, we propose that cross-device transformation of OCTA data leveraging unpaired image-to-image translation techniques may be a promising approach. It should be noted, however, that this commentary does not report actual implementation of these experiments. Nevertheless, we can think of a few challenges ahead in that direction, as described below.

### Practical challenges

First, although data from different OCTA devices are not directly interchangeable [3, 5–7, 36], this may not necessarily imply that the inter-domain variations are very large. Indeed, there may be many shared features among different OCTA domains, and as a downside, such overlapping features can interfere with competent learning of the model – because it tries to learn features that do not help transform one domain to another (see Fig. 2.b). To overcome this, as suggested by Manakov et al., using a shared discriminator may accelerate the training and improve the performance [25].

The second challenge is that training numerous models using the original Cycle-GAN architecture requires too much computational power, especially given the number of commercialized OCTA machines. For example, for 5 devices, we will need 20 generators to perform all direct cross-device translations (see Fig. 2.c).

We think one possible solution is experimenting with alternative architectures that incorporate an intermediate domain with feature distributions lying between other domains (e.g., device C in Fig. 2.a). Hypothetically, this maneuver may enable indirect cross-domain translation with significantly reduced computational costs (see Fig. 2.c). Of note, the accuracy and efficiency of such indirect translation methods remain unclear. Alternatively, multiple-domain variants of Cycle-GAN may also help, for example, StarGAN, which was introduced by Choi and colleagues in 2018 [37]. StarGAN works with only one generator and one discriminator, which does not only discern synthetic from actual images but also identifies the domain to which an image belongs [37].

The third challenge lies in the intrinsic difficulty of training GANs. To elaborate, the ideal outcome of the rivalry between the generator and the discriminator is achieving a Nash equilibrium between the models where they both excel at their tasks and cannot improve further – i.e., the global optima. However, the training process often encounters situations where the generator fails to grasp the full range of features and may produce identical, low-quality images over and over – i.e., ‘mode collapse’. This can occur when the generator has been provided with – or focused on – only a limited subset of the real-world feature distribution. Another reason may be the discriminator outpacing the generator in feature learning, consequently perceiving every synthesized image as fake, even if it is better than the last iteration. This hinders the generator’s further improvement [38]. Therefore, optimization of Cycle-GANs often proves difficult and burdensome. On the other hand, the non-adversarial nature of DDPMs allows a more stable training process, where mode collapse may not be a problem [15].

The Fourth challenge is that domain adaptation across different devices alone cannot account for variations in the subject populations. Thus, large datasets representing a broad spectrum of healthy and diseased eyes are still necessary. Overall, technical aspects aside, acquiring representative OCTA data from different devices necessitates large-scale collaborative efforts by multiple centers.

## Conclusion

We are overwhelmed by the recent advancements in retinal imaging technologies and the rapid progress of advanced, efficient DL-based domain adaptation techniques. In light of this, we propose that image-to-image translation methods have the potential to enhance the generalizability and comparability of OCTA data from different devices or centers. We strongly encourage interdisciplinary efforts in this direction, emphasizing the importance of ensuring that experts in the field are aware of such continuous advancements in unpaired domain adaptation approaches. Such efforts will pave the way for more efficient pooled analysis of OCTA findings from various centers, which will result in more conclusive interpretations of the existing yet heterogeneous data. Last but not least, this approach may also enable wider applicability of current or future ML/DL models trained on smaller datasets from a limited number of OCTA device types.

## Data Availability

Not applicable.

## Abbreviations

AI: artificial intelligence
CFP: color fundus photograph
CT: computed tomography
Cycle-GAN: cycle-consistent Generative Adversarial Network
DA: domain adaptation
DDPM: Denoising Diffusion Probabilistic Model
DL: deep learning
DR: diabetic retinopathy
FAZ: foveal avascular zone
GAN: Generative Adversarial Network
ML: machine learning
MRI: magnetic resonance imaging
OCT: optical coherence tomography
OCTA: optical coherence tomography angiography
UNIT-DDPM: Unpaired Image Translation with Denoising Diffusion Probabilistic Model
